# Waste cooking oil processing over cobalt aluminate nanoparticles for liquid biofuel hydrocarbons production

**DOI:** 10.1038/s41598-023-30828-0

**Published:** 2023-03-08

**Authors:** M. A. Ibrahim, R. El-Araby, Elham Abdelkader, Mohamed El Saied, A. M. Abdelsalam, E. H. Ismail

**Affiliations:** 1 Chemistry Department, Ain Shams University Chemistry Faculty of Science, Cairo, Egypt; 2 Chemical Engineering and Pilot Plant Department, National Research Centre, Cairo, Egypt; 3 Egyptian Petroleum Research Institute, Cairo, Egypt; 4 Misr Petroleum Company, Cairo, Egypt

**Keywords:** Energy science and technology, Engineering

## Abstract

The catalytic conversion of waste cooking oil (WCO) was carried out over a synthetic nano catalyst of cobalt aluminate (CoAl_2_O_4_) to produce biofuel range fractions. A precipitation method was used to create a nanoparticle catalyst, which was then examined using field-emission scanning electron microscopy, X-ray diffraction, energy dispersive X-ray, nitrogen adsorption measurements, high-resolution transmission electron Microscopy (HRTEM), infrared spectroscopy, while a gas chromatography-mass spectrometer (GC–MS) was used to analyze the chemical construction of the liquid biofuel. A range of experimental temperatures was looked at including 350, 375, 400, 425, and 450 °C; hydrogen pressure of 50, 2.5, and 5.0 MPa; and liquid hour space velocity (LHSV) of 1, 2.5, and 5 h^−1^. As temperature, pressure, and liquid hourly space velocity increased, the amount of bio-jet and biodiesel fractional products decreased, while liquid light fraction hydrocarbons increased. 93% optimum conversion of waste cooking oil over CoAl_2_O_4_ nano-particles was achieved at 400 °C, 50 bar, and 1 h^−1^ (LHSV) as 20% yield of bio-jet range,16% gasoline, and 53% biodiesel. According to the product analysis, catalytic hydrocracking of WCO resulted in fuels with chemical and physical characteristics that were on par with those required for fuels derived from petroleum. The study's findings demonstrated the nano cobalt aluminate catalyst's high performance in a catalytic cracking process, which resulted in a WCO to biofuel conversion ratio that was greater than 90%. In this study, we looked at cobalt aluminate nanoparticles as a less complex and expensive alternative to traditional zeolite catalysts for the catalytic cracking process used to produce biofuel and thus can be manufactured locally, which saves the cost of imports for us as a developing country.

## Introduction

The scarcity of conventional petroleum fuels, combined with environmental deterioration, has induced to seek alternative energy sources and develop biomass conversion technology to obtain alternative products to substitute petroleum fuels, and then come up with a strategy to tackle the issues of the predicted scarcity of abundance. Biofuels may be the optimum alternative to fossil fuels^1–3^, also they are described as long-term energy sources that contain a significant amount of energy saved within them by living organisms if either animals or plants. It is one of the most promising sources of renewable energy when compared to other natural resources like coal and petroleum, natural gas, or nuclear fuel. It also differs from fossil fuels in terms of the low cost of production and positive environmental friendliness, both of which contribute to the reduction of climate change^3–5^.

The key drawback of first-generation biofuels is that they are made from biomass, which is also a food source^8^. Several more researchers, on the other hand, wanted to look into converting WCO into biofuels as an alternate fuel. The abundance of massive WCO production, projected at thousands of tons per year, assisted in its application^6–8^. Because they share physicochemical characteristics with derived oil fuels, green diesel and other hydrocarbon biofuels created through the catalytic deoxygenation of vegetable oils are a viable alternative to mineral diesel. The catalyst, hydrogen pressure, temperature, and type of vegetable oil used can all have an impact on the type of biofuel produced and its characteristics^9^. The resulting green diesel can be used as a stand-alone fuel in traditional diesel engines or combined with petroleum diesel. The implementation of effective solid catalysts for vegetable oil hydroprocessing is rapidly growing. Some researchers have researched on metal-to-support electronic contact on catalytic activity and hydrodeoxygenation selectivity in vegetable oil hydroprocessing^9,10^. The hydrocracking process employs catalytic cracking and hydrogenation to produce lighter fraction products from heavy fractions. This high-temperature and high-pressure process necessitate the use of a catalyst and hydrogen^11,12^. Hydrocracking is part of the hydro-treatment process. Decarboxylation (DCO2), decarbonylation (DCO), and hydrodeoxygenation (HDO) are common hydrotreatment reactions that take place during the hydrocracking process. Depending on whether a catalyst is present, the decarbonylation process is further divided into two categories: catalytic reactions and thermal reactions^13^. Side effects have been reported as the temperature rises. These side effects will reduce yield value, and the industry is heavily focused on prevention. Two common side reactions are methanation and reverse water gas shift. As the temperature rises during the decarboxylation process, the first side reaction (reverse water gas shift) occurs, accompanied by methanation until a certain temperature is reached. Methanation is the only possible side reaction during the decarbonylation process^14,15^. To convert vegetable oils into biofuel in the presence of hydrogen, hydrocracking, a hybrid of catalytic cracking and hydrogenation, requires high temperatures (300–400 °C), high hydrogen pressure, an active catalyst, and more energy. The hydrocracking products, such as gasoline and kerosene, had better oxidation stability and higher cetane numbers^16^.

**Figure 1:**
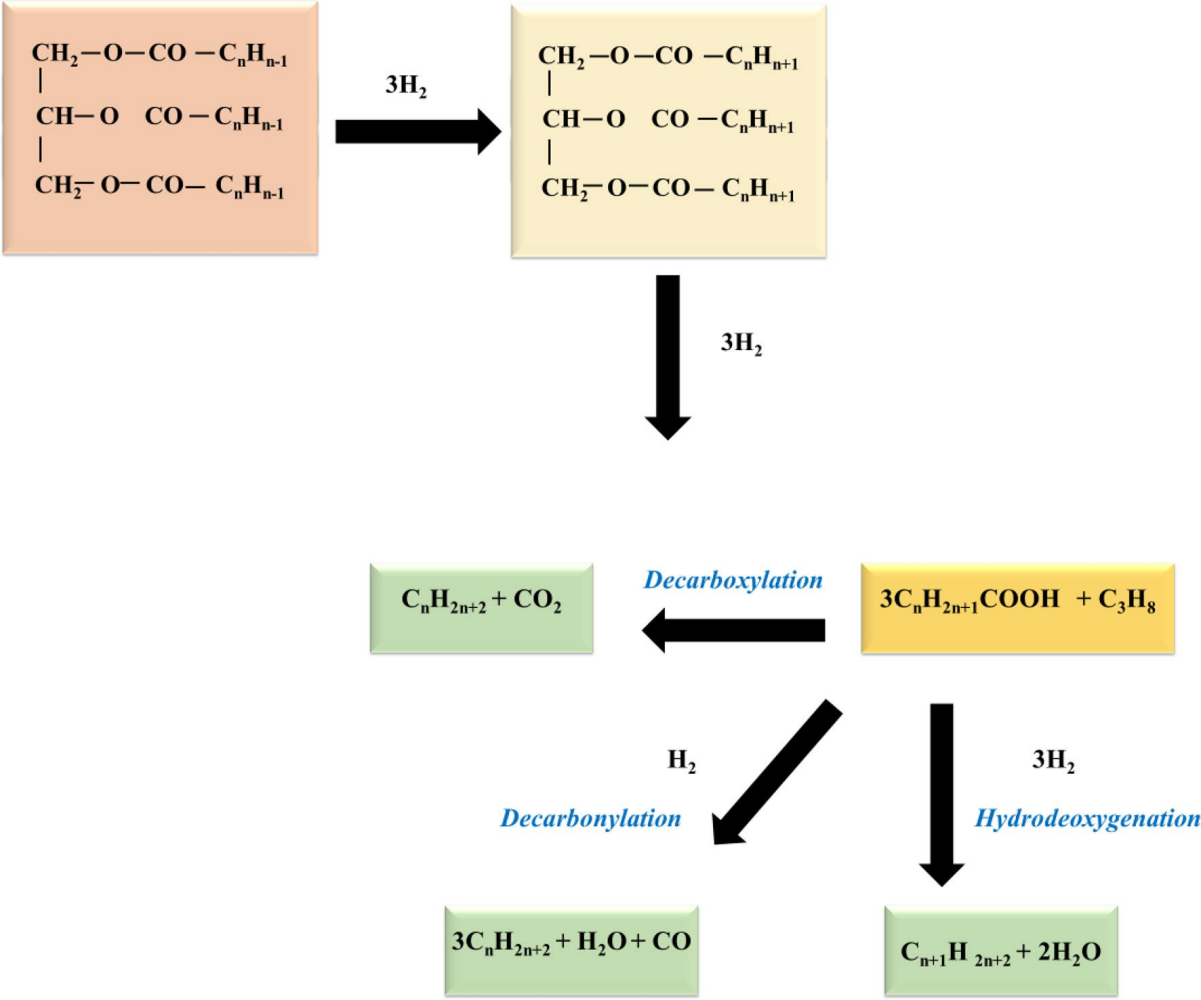
Hydro-conversion of a triglyceride.

Decarbonylation and decarboxylation both remove oxygen; however, Oxygen is decarbonylated to produce carbon monoxide and water, whilst decarboxylation produces carbon dioxide. The hydrogenation step is performed out in batch and continuous reactors, with the reaction temperature and hydrogen pressure determining the efficiency, Fig. 1^15,17^. The use of two distinct types of catalytic sites to catalyze different steps in the reaction system is required for hydrocracking reaction progression via a dual functional mechanism. The metallic function enhances dehydrogenation, hydrogenation, and inappropriate hydrogenolysis, whereas the acidic function promotes cracking and isomerization. Cracking occurs when high-acidity catalysts deactivate. It is necessary to maintain a proper balance between acid and metal site density during the creation of effective hydrocracking catalyst performance^18,19^.

The development of high selectivity, long lifetime catalysts has the potential to lower biofuel production costs while increasing product yield. Alumina- and zeolite-based catalysts are typically employed for upgrading both vegetable oil and bio-oil. Metal catalysts supported on SiO_2_/SO_4_,18 Ni-Mo supported catalyst,6 Co-Mo metal impregnated natural zeolite,9 bimetal and trioic acid supported on SBA-15 catalyst,19 ZSM5 zeolite,20 zeolite-Al_2_O_3_ composites supported NiMo catalyst^19–21^ which are used in the catalytic hydrocracking of vegetable oil to produce biofuels like bio-gasoline and bio-aviation fuel.

HZSM-5 (Hydrogen Zeolite Socony Mobiles Number 5) performed well, having the highest yield of gaseous products when compared to acidic catalysts for cracking palm oil because its pore size is similar to that of a triglyceride molecule^22,23^.

Due to its numerous uses as an inorganic ceramic blue pigment for coloring plastics, paint, fiber, paper, rubber, phosphor, glass, cement, glazes, ceramic bodies, and porcelain enamels, cobalt aluminate has drawn attention. Cobalt aluminate is also used in other heterogeneous catalysis processes, such as the selective catalytic reduction of NOx with hydrocarbons and the reforming of methane with carbon dioxide. According to certain experts, nanocrystalline cobalt aluminate has a great deal of potential for usage in UV-light photocatalytic applications. The created cobalt aluminate nanoparticles were employed as photocatalysts to remove red textile dye from synthetic effluent by photocatalytic oxidation. It serves also as a humidity sensor as well.

Among the previous uses of cobalt aluminate, the present study aimed to prepare cobalt aluminate nanoparticles as a simpler and less expensive catalyst than conventional zeolite catalysts used to convert low-cost WCO feedstock to liquid hydrocarbon ranges of biogasoline, biodiesel, and biojet using hydrocracking in a continuous high-pressure reactor under an optimized reaction temperature, hydrogen pressure, and liquid hourly space velocity , which allows us, as a developing country, to produce biofuel for from local raw materials instead of expensive imports.

## Experimental work

### Feedstocks

In this study, the waste cooking oil was obtained as liquid waste from local Cairo eateries. The sample was heated to 60 °C and filtered under vacuum in a press filter to obtain clear and clean spent fry oil, then dehydrated up overnight using anhydrous sodium sulfate, and finally filtered under vacuum once more.

### Synthesis of (CoAl_2_O_4_) nanoparticles catalyst

The co-precipitation method^2,24^ was used to prepare the catalyst of cobalt aluminate nanoparticles from metal nitrates, with ammonia serving as a precipitating agent. After dissolving Al (NO_3_)_3_9H_2_O in 10 ml of distilled water, 20 mL of Co (NO_3_)_2_ 6H_2_O (20 mmol) was added to the solution (40 mmol). The correct amount of aqueous ammonia solution (25 weight percent) was added to the aforementioned solution, and the mixture was stirred until complete precipitation occurred at a pH of 8 to 9. The precipitate was filtered, distilled water washed, and dried. The dry precipitate was calcined at 600 C for 4 h to produce the CoAl_2_O_4_ nanoparticles.

### Catalytic activity testing for bio-fuel production

**Figure 2:**
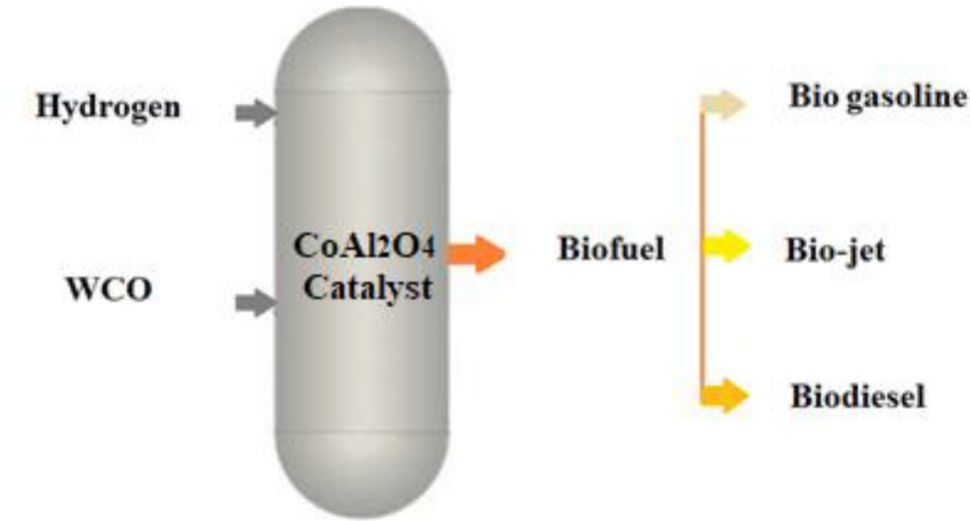
Graphical Summary of The Catalytic Activity Testing.

Figure 2 shows the graphical summary of the catalytic activity testing for bio-fuel production was conducted by a Continuous High-Pressure Reactor.

#### Continuous high-pressure reactor

**Figure 3:**
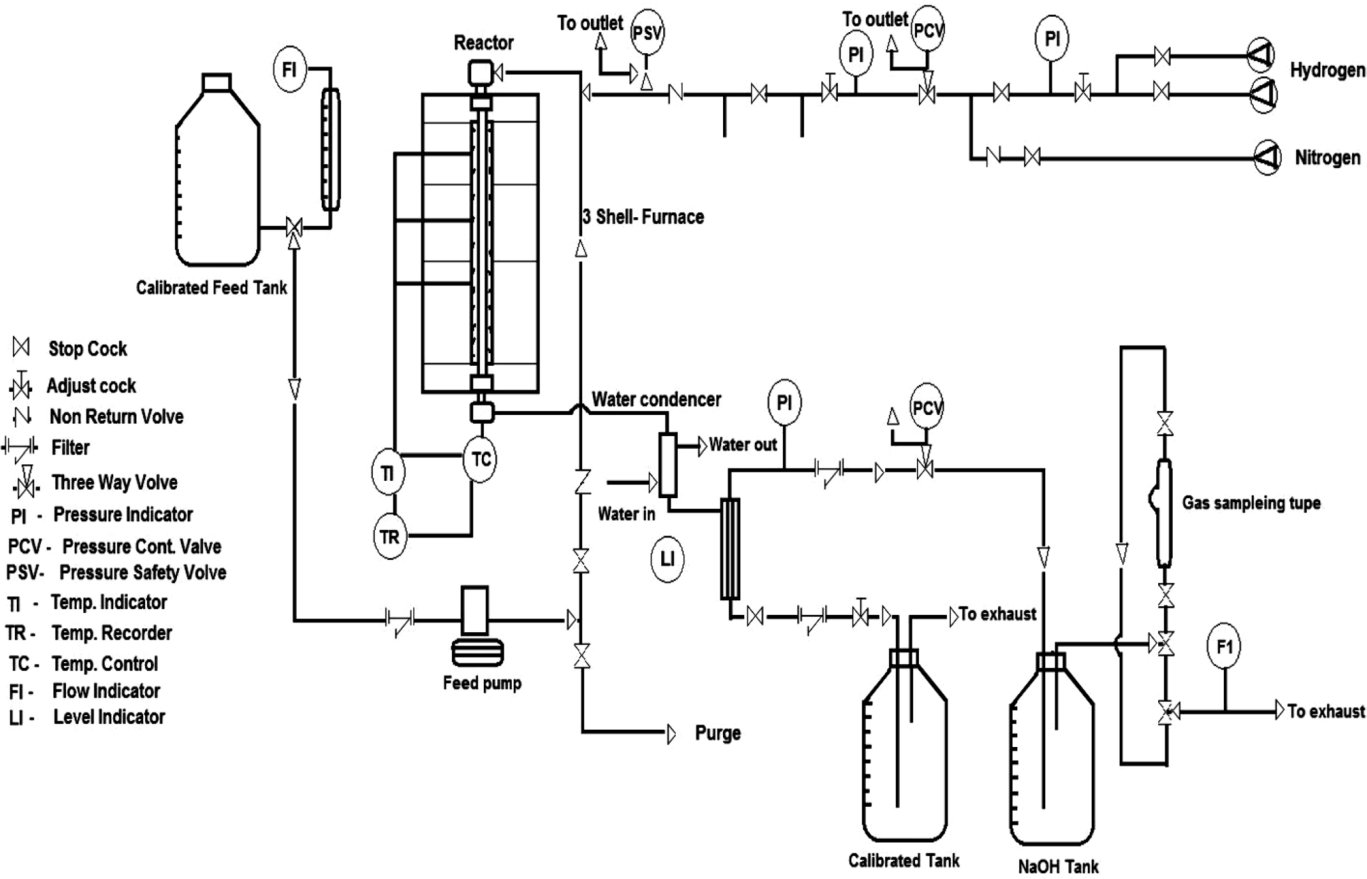
High-Pressure Micro-Reactor Unit.

A series of experiments were carried out to explore the effect of various operating factors on the quality and quantity of the obtained products by using the synthesized cobalt aluminate nanoparticle catalyst. In a continuous high-pressure micro-reactor unit, reactions were conducted as shown in Fig. 3. The main component of the unit is a 50 cm vertical tubular stainless-steel reactor with internal and external diameters of 19 and 27 mm, respectively. It is divided into three main sections, each of which is connected to its heating element and temperature control. The reactor was loaded in the following manner from top to bottom: 13 cm (30 mg) of the catalyst under investigation in the middle zone, and another 17 cm of porcelain beads (preheating zone). A H_2_ cylinder delivered hydrogen gas to the unit, while a piston pump with an adjustment knob was used to pump liquid feed to the reactor's top.

For the investigated oil, the hydrotreating and hydrocracking reactions were carried out under various operating conditions. The reaction temperature, hydrogen pressure, and feedstock liquid hourly space velocity are the main operating parameters of hydroprocessing reactions. The effect of each variable on the process was investigated while maintaining the H_2_/WCO ratio constant at 600 V/V and other variables constant. The following experimental factors were investigated:

- Temperature: 350,375, 400, 425 and 450 °C,
- Hydrogen Pressure: 50, 2.5, and 5.0 MPa,
- Liquid Hour Space Velocity: 1, 2.5, and 5 h^-1.^

The unit was flushed with hydrogen gas before the feedstock oil was fed by the pump, and it was then kept under hydrogen gas pressure to check for leaks. The operating conditions in the hydro-conversion runs are then adjusted as needed. The feed was pumped after reaching a steady state, and the reactor effluent was allowed to cool in a water condenser before being separated into liquid and gas phases. The liquid product (organic and aqueous fractions) was gathered and measured in a receiver, while the gases were diverted to the outlet using a gas meter. The contaminated water from the liquid yield was removed after 24 h of settling in a separating funnel. To remove any remaining water, anhydrous sodium sulfate was added to the organic product. Organic products, as well as crude oils, were analyzed and evaluated. The unconverted vegetable oil was separated from the organic product using an atmospheric distillation unit. The residue that remained after distillation was known as residual and/or unconverted oil. Furthermore, fractional distillation was used to separate the produced biofuel into three fractions based on their boiling points, which were blended in different ratios: biogasoline and a light fraction (90–150 °C), biokerosene (150–270 °C), and biodiesel (270–350 °C). All of these products were estimated and characterized by the various ASTM standards.

#### Fractional distillation

For petroleum products and middle distillates, the atmospheric distillation method in ASTM D-86 was applied. This method can be used to determine the boiling range of numerous different hydrocarbon distillates, such as gasoline with or without oxygenates, diesel, and other light and middle distillates ^24,25^. Fractional distillation was used to separate the product mixtures produced by catalytic thermal cracking, and the percentage of the volumes was calculated at various (BP) boiling points between 50 and 350 °C. The distilled fractions' yield , as well as the bottom residual product, were calculated using Eq. (1)^26^**.**

1 $${\text{Distilled Fraction }}\left( {{\text{wt}}.\% } \right) = {\text{mass of distilled fraction}}/{\text{mass of organic liquid product}}$$

### Characterization of Synthesized Nano CoAl_2_O_4_ Catalyst

The investigated catalysts were evaluated employing the following techniques:

#### Energy dispersive X ray analysis (EDX)

Using the EDX method, it was possible to calculate the weight percentage of each element present in the parent Nano CoAl_2_O_4_ catalyst under investigation. This technique involves hitting the sample under examination with electrons, which leaves a void inside the atoms of the sample. Higher energy electrons from the atoms' outer shell then fill this void.

#### X-ray analysis

The advanced X-ray diffractogram X' Pert PRO was used to determine the x-ray diffraction (XRD) patterns of the catalysts using Cu-Ka radiation (= 1.5418) at (2 = 7–70), a scan speed of 0.04/10 s.

#### Nitrogen adsorption measurements

Using a NOVA 3200 apparatus from the USA, N_2_ adsorption studies were used to characterize the surface area and surface parameters of the solid materials at −196 °C. The samples had previously been outguessed for five hours at 300 °C and vacuum (10–4 Torr). By using the standard Burunuer, Emmett, and Teller (BET) procedure, surface areas (SBET) were calculated from the adsorption branch. The Barret- Joyner- Halenda (BJH) method was used to obtain the pore size distributions. Additionally, estimated pore volumes for each catalyst used were made.

#### High-resolution transmission electron microscopy (HRTEM) images

All catalyst samples were examined for morphology and particle size using high-resolution transmission electron microscopy (TEM) equipment (JEOL JEM 2100 Model, Japan), which was attached with (EDX) Oxford X- Max. A drop of the solution was applied to a carbon-coated Cu TEM grid to prepare the TEM samples by ultrasonically dispersing a diluted particle-ethanol colloidal mixture for 30 min.

#### Scanning electron microscope (SEM)

The shape and surface morphology of CoAl_2_O_4_ nanoparticles were studied using HR-SEM.

#### Fourier transform infra-red (FTIR) spectroscopy

Nicoletis- LOFT- IR, Nicolet IS-10 Fourier Transform Infra-Red (FTIR) analysis was used to examine the structural characteristics of the catalysts. The spectra were captured in transmittance mode between 500 and 4000 cm-1.

#### NH_3_-TPD analysis

NH_3_-TPD is a common analysis used to determine the total acidity of solids. The amount of ammonia desorbed is taken as a measure of the number of acid sites, the desorption temperature indicates the strength of acid sites^27^.

#### Products analyses

Various standard ASTM procedures were used to test the biodiesel and biojet fractions derived from the atmospheric distillation process of the generated organic liquid.

## Results and discussion

### Catalyst characterization

#### Energy dispersive X ray analysis (EDX)

**Figure 4:**
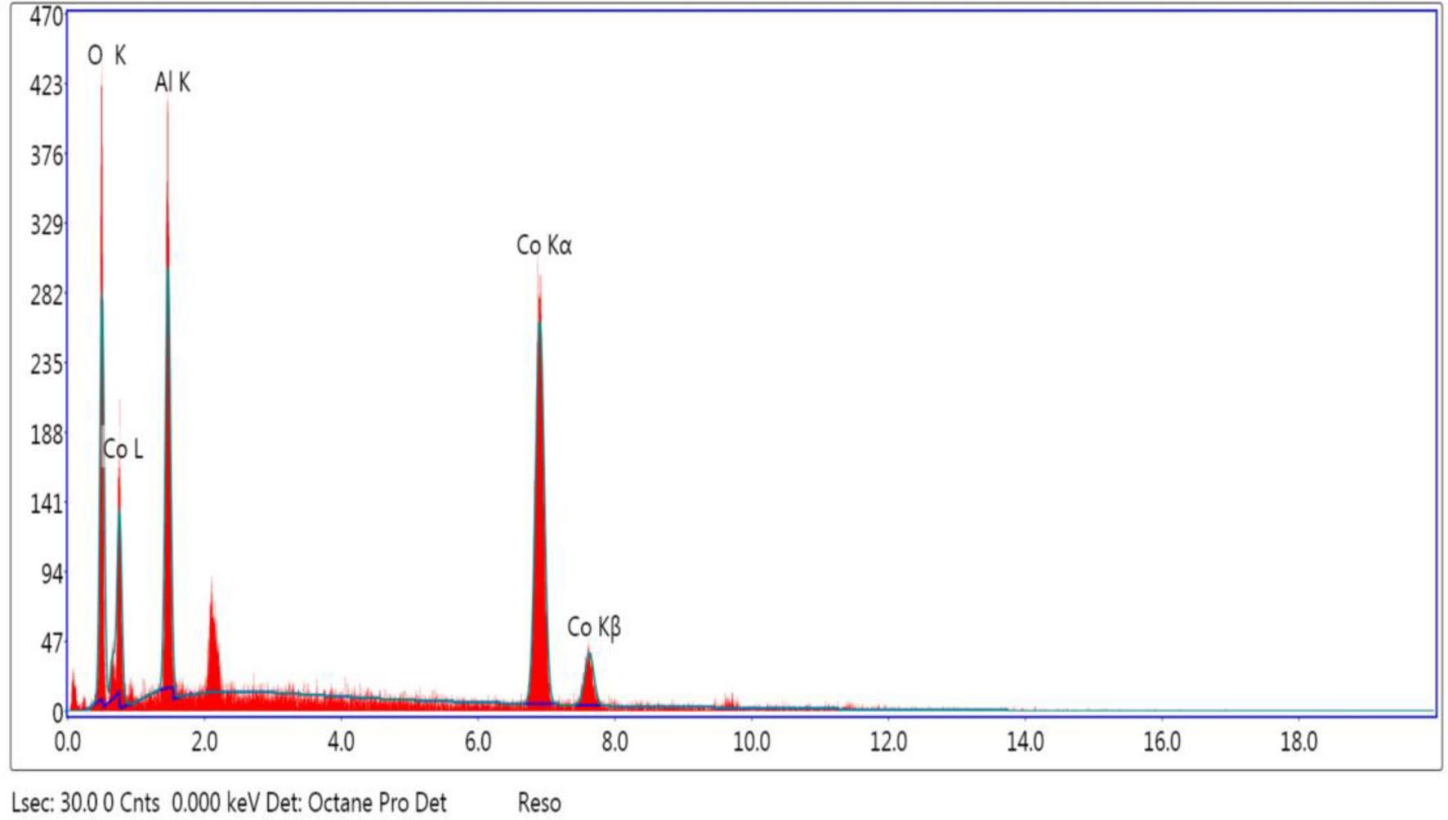
EDX Spectrum of The Parent Nano CoAl_2_O_4_ Catalyst.

The idea of EDX characterization of catalysts is to study the chemical elements or properties of the specimens. This characterization is based on studies of the interaction of various X-ray excitations with specimens. Each element has a distinct atomic structure and is identifiable by X-rays due to its atomic structure^28^. The number of X-rays that were produced depended on the type of atom and was caused by the conversion of these higher energy electrons to lower energy shells. As a result, depending on its concentration in the sample under test, each atom will have a distinct peak with a particular height in the EDX spectrum. The typical EDX spectra obtained for the parent CoAl_2_O_4_ catalyst are represented in Table 1 and Fig. 4.

**Table 1:** EDX Analysis for The Parent Nano CoAl_2_O_4_ Catalyst.

Element	Weight %	Atomic %	Net Int	Error %
O	21.84	44.5	140.96	8.5
Al	18.73	22.62	178.65	9.02
Co	59.44	32.88	271.68	3.2

Accordingly, there is a large density of active sites represented in cobalt, which increases the activity and efficiency of the catalyst towards the reaction^29^.

#### X-ray diffraction analysis (XRD)

**Figure 5:**
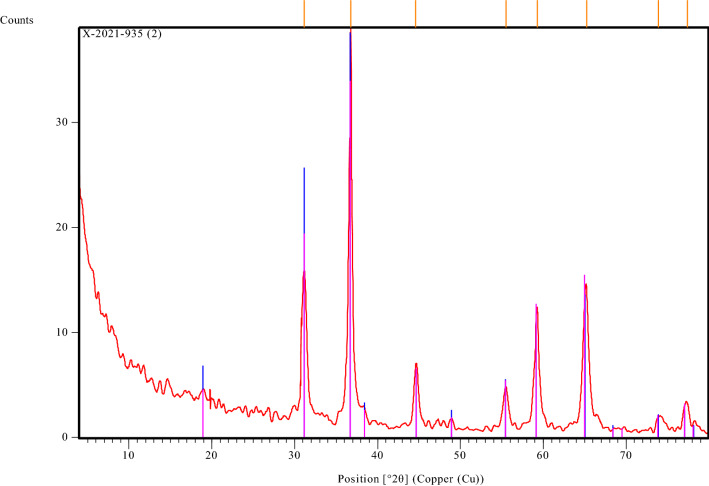
X-Ray Diffraction of Nano CoAl_2_O_4_ Catalyst.

Figure 5 depicts the powder X-ray diffraction patterns of the prepared catalyst. According to (ICDD card No. 00-004-0160), the sample has crystallized in a single phase with a spinel structure, space group Fd-3 m, and the major spinel phase that is indexed cubic phase of CoAl_2_O_4_ structure with color deep blue. The literature supports this^30,31^. The (210), (315), (402), (334), (419), (516), (443), (622), and (530) planes of CoAl_2_O_4_ can be attributed, respectively, to the observed diffraction peaks at 2, which are 36.79, 44.64, 55.52, 59.33, 65.25, 73.91, and 77.42. Using the Scherrer formula to determine the line broadening of the (220), (311), (511), and (440) peaks, the mean grain size of the sample was quantitatively determined to be 14, 18, and 25 nm, respectively. Finally, it is clear from the XRD pattern that the sample is pure crystal, which is compatible with the literature, and this indicates the successful preparation of this catalyst, which is considered a promising material in the production of biofuels from WCO.

#### Nitrogen adsorption measurements

**Figure 6:**
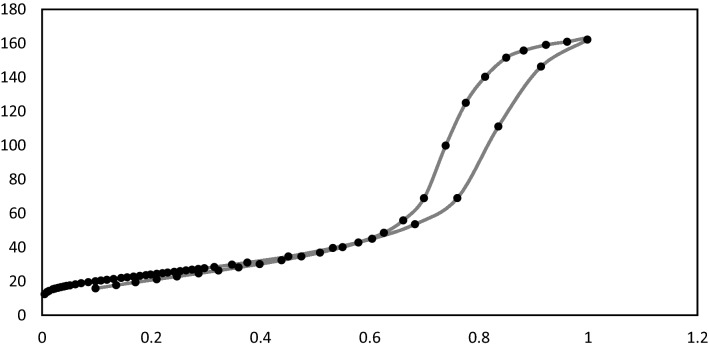
N_2_ Adsorption Desorption Isotherm of Nano CoAl_2_O_4_ Catalyst.

Figure 6 illustrates the nitrogen adsorption/desorption isotherms and pore size distributions of the prepared sample. (i) Monolayer-Multilayer Adsorption, (ii) Capillary Condensation, and (iii) Multilayer Adsorption on the Outer Particle Surfaces are three distinct regions of the Adsorption Isotherm. As stated by the International Union of Pure and Application Chemistry's classification, the prepared sample appears to have a type IV isotherm (IUPAC)^32,33^**.** The CoAl_2_O_4_ catalyst has a BET surface area of 86.914 m^2^/g, a pore diameter of 7.602 nm, and a pore volume of 0.274 cm^3^/g, according to data in Table 2. One of the most important features of this catalyst is the existence of pores with a diameter of 7.2 nm, and this helps to spread the active sites on all parts of the prepared material and also inside these holes. These data suggest that the relatively large pores and high surface area of the sample are beneficial to the formation of active sites, providing excellent catalytic activity^28^**.** The presence of this size of holes through the catalyst helps to facilitate the entry of reactants into the pores, which contain active sites, and thus increases the production yield.

**Table 2:** Texture Properties of CoAl_2_O_4_ Catalyst.

Catalyst	SSA/m^2^/g	Pore diameter/nm	Pore size distribution/ccg
Co/Zn-Al_2_O_4_	86.914	7.602	0.274

#### High-resolution transmission electron microscopy (HRTEM)

**Figure 7:**
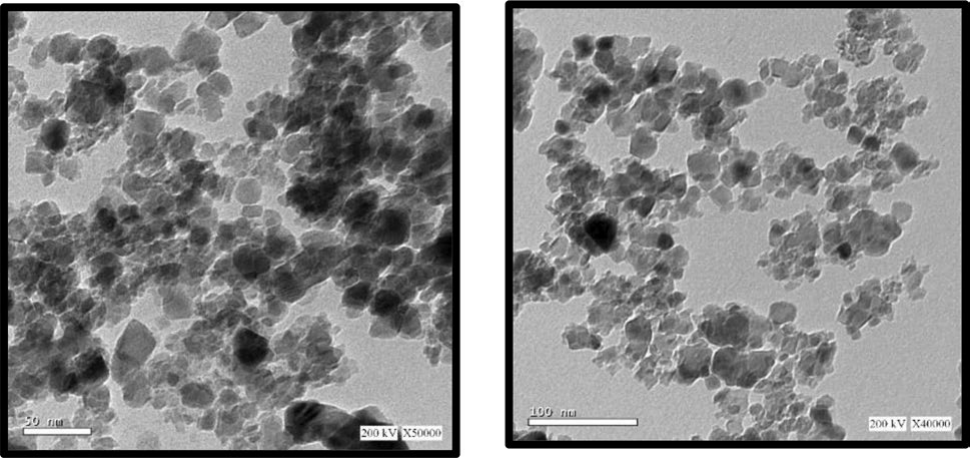
HRTEM of Nano CoAl_2_O_4_ Catalyst.

High-resolution transmission electron microscopy (HRTEM) produces direct images of the atomic structure of samples, allowing for direct information about the crystallographic structure of materials to be obtained from images^34^**.** Images with high phase contrast as small as a crystal cell can be obtained. The transmission micrographs for the CoAl_2_O_4_ sample are shown in Fig. 7. The image demonstrates that the cobalt aluminate particles are uniform, nanoaggregate, and less than 50 nm in size. As shown in the figure, the cobalt is homogeneous in distribution, which means that the active sites are spread throughout all parts of the sample, and this reduces reaction time and increases the production of yield^23,35^.

#### Scanning electron microscope (SEM)

**Figure 8:**
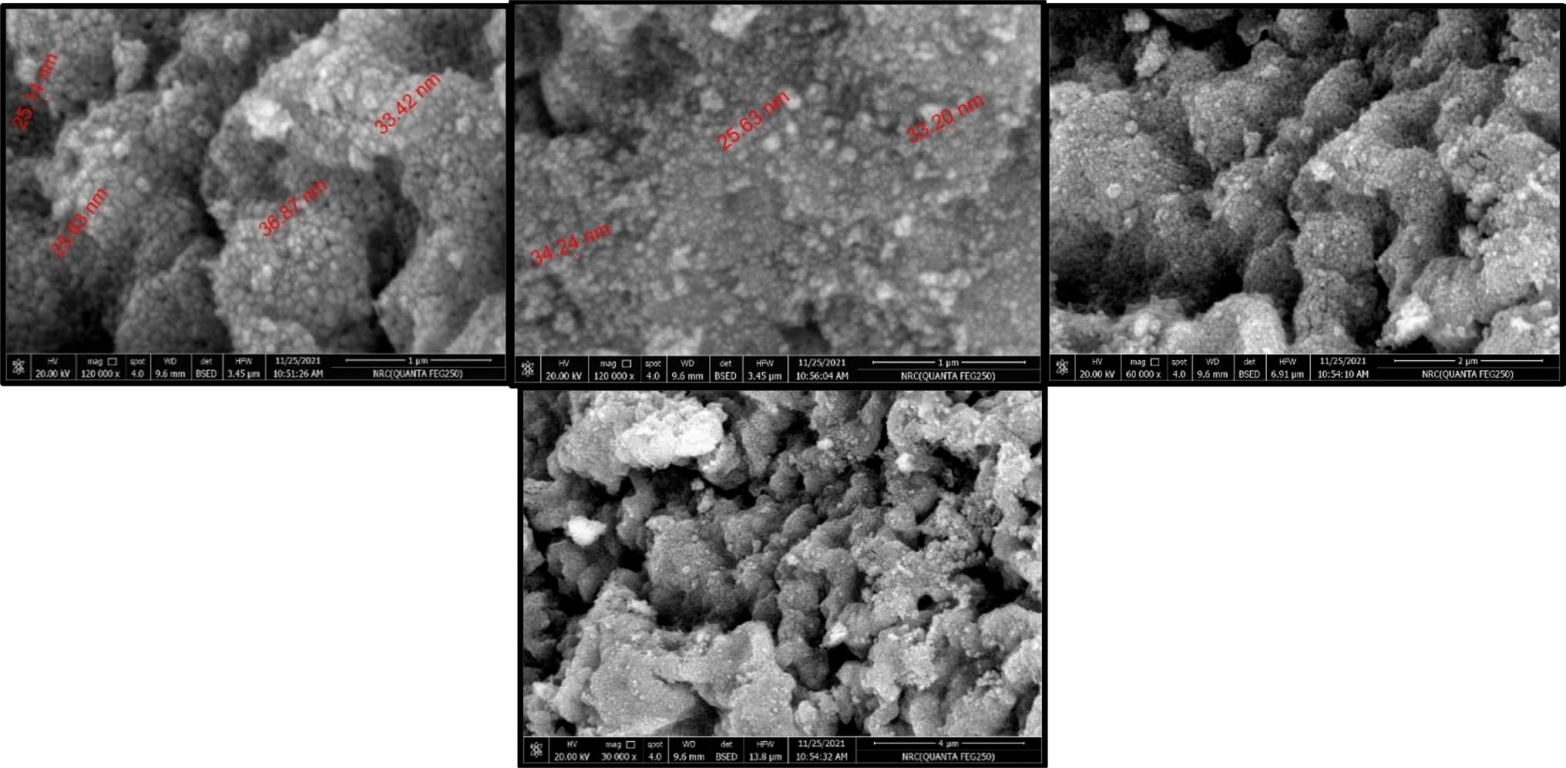
Scanning electron microscopy (SEM) images of CoAl_2_O_4_ catalysts.

SEM is an excellent method for determining the purity, level of aggregation, degree of dispersion, and homogeneity of nanoparticles^26,36^. Therefore, HR-SEM was used to examine the surface morphology and shape of CoAl_2_O_4_ nanoparticles^23,37^. Cobalt aluminate nanoparticles were found to be uniformly distributed and homogeneous in HR-SEM images, as shown in Fig. 8. The SEM images demonstrate that there is little aggregate formation and that the particle sizes range from 25.14 to 25.63 nm and 33.42 to 36.87 nm.

#### Fourier transform infrared spectroscopy (FTIR)

**Figure 9:**
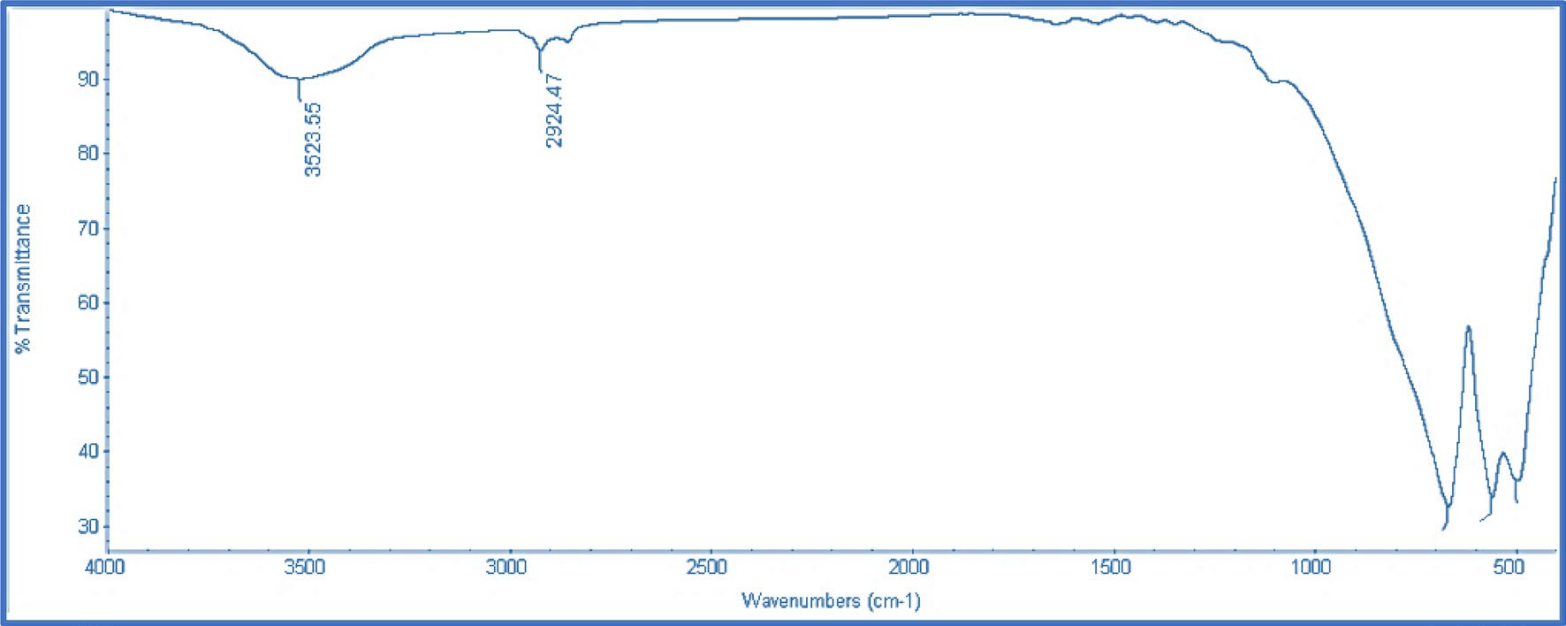
FT-IR of Prepared CoAl_2_O_4_ Catalyst.

The FT-IR spectrum of the CoAl_2_O_4_ nanoparticles produced by calcining the precursor at 600 C for 4 h is shown in Figure 9, which shared a broad band near 3500 cm 1 and near 1600 cm 1 due to the OH stretching vibrations and deformative vibrations of water molecules, respectively. The Al–O stretching and O–Al–O bending vibrations of the AlO6 groups in the spinel-type CoAl_2_O_4_ structure, respectively, are related to the two strong bands that were seen below 1000 cm1, which are located around 666 and 559 cm^-1^^23^.

This outcome is consistent with the outcome of the XRD analysis. It should be noted that the method of CoAl_2_O_4_ preparation affects the number and shape of its FT-IR bands. It displays two or three bands between 450 and 700 cm^-1^. The large dispersion of cobalt nanoparticles may increase the probability of produced n-paraffin being collected on active sites^38^**.**

#### NH_3_ TPD analysis

**Figure 10:**
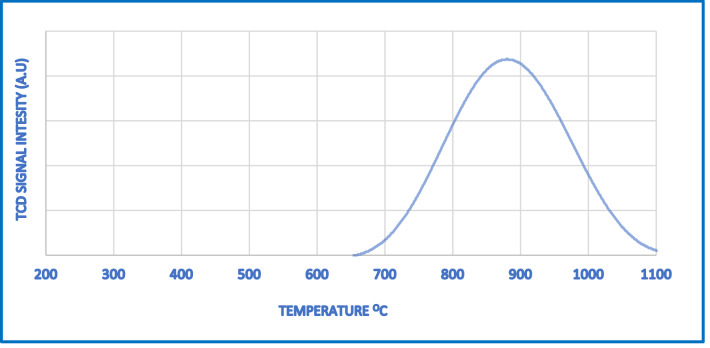
NH_3_-TPD of CoAl_2_O_3_ catalyst.

It can be seen from this Fig. 10 that the desorption of cobalt aluminate consists of a distinct unique peak concentrated at 800 °C. The strong peak observed for CoAl_2_O_3_ which corresponds to the structural Brønsted acid sites generated by the overlap of Co^2+^ for Al^3+^. These structural Brønsted acid sites are generated may be due to the charge difference between the Al^3+^ ion and the Co^3+^ ion, which replaced it in the process of substitution, because of which the lattice acquires a one-electron charge that must be compensated for by an extra framework cation for the stabilization of the matrix^28,29^**.** On the other hand, this strong peak may form because of the presence of tricoordinated aluminum or octahedral aluminum oxide or oxyhydroxide species present in the matrix due to incomplete condensation of the network^30^. It is also illustrated in the literature that framework cobalt can also act as a Lewis acid center in cobalt-containing molecular sieves^31^**.** Indeed, the inclusion and great dispersion of metal species significantly increases the acid sites because metal species generate new Lewis acid sites for NH3 chemisorption^39^.

#### Catalytic cracking over CoAl_2_O_4_ nanoparticles

The WCO cracking was performed over the synthesized CoAl_2_O_4_ Nanoparticles at varied reaction conditions of temperature, pressure, and liquid hourly space velocity. The organic liquid product was fractionated using atmospheric pressure distillation. Atmospheric distillation is a simple, mature separation technique that presents itself as a unique economically feasible and scalable way to separate the complicated liquid product composition into chemical fractions based on vapor pressure variations^40^.

#### Effect of reaction temperature

At varied reaction temperatures of 350,400 and 450 °C, the cracking process was performed with aid of the synthesized CoAl_2_O_4_ Nanoparticles then, the organic liquid product was subjected to atmospheric fractional distillation to obtain three major fractions within the distillation temperature ranges of light fraction of 90–150 °C (biogasoline), 150–270 °C (biojet), and 270–340 °C (biojet) (biodiesel). The distillation temperature intervals were reported to produce hydrocarbon fractions with characteristics identical to certain petroleum products ^26^. In terms of percent conversion and yield of each obtained fraction from the distillation stage, the effect of reaction temperature was observed. It was suggested that the WCO would first crack thermally and catalytically on the catalyst's outer surface to produce heavy hydrocarbons and oxygen, which would then crack within the inner pore structure of CoAl_2_O_4_ to produce light alkenes, alkanes, water, carbon dioxide, and carbon monoxide. Different reactions may dominate in nanoparticles at various reaction temperatures, which could help to explain how uneven temperature affects conversion. Due to the higher rate of cracking, Table 3 demonstrates that the conversion of WCO rises steadily as the reaction temperature rises. The highest conversion occurred at 450 °C, 50 bar of hydrogen pressure, and 1 h-1, with a 95.7 weight percent rate. The same results were previously confirmed by similar trends^41–43^**.** In general, it is clear from the distillation process results that the yield of the light and bio-jet fractions increased as reaction temperature increased; the highest yield of bio-jet reached 28%, while the highest yield of biodiesel was obtained at 400 °C in the same LHSV of 1 h^-1^, as shown in Table 3.

**Table 3:**
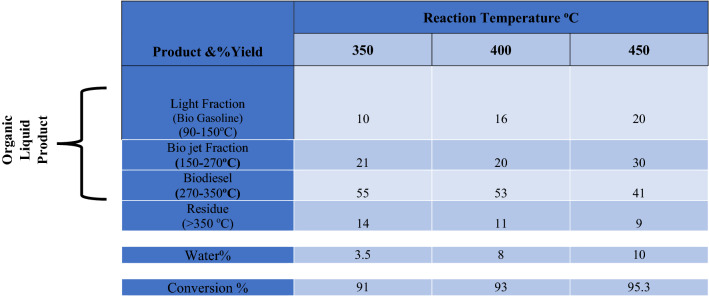
Effect of Reaction Temperature on Hydro-conversion of WCO over CoAl_2_O_4_ Nanoparticles. (Pressure 50 bar, LHSV: 1 h^-1^).

#### Effect of operating pressure

The starting hydrogen pressure contributed to the hydrocracking process during the reaction^38^. Another crucial element for the hydrogenation process is hydrogen pressure. To increase the yield of the hydrodeoxygenation reaction, which produces hydrocarbons, the higher hydrogen pressure is necessary^44^.

Several experiments were carried out in this study at three different pressures, namely 30 bar, 50 bar, and 70 bar Table 4. Hydrotreating WCO feedstocks has demonstrated that raising the hydrogen pressure in the system raises the concentration of hydrogen in the liquid mixture, favoring hydrogenation and hydrogenolysis reactions. Additionally, since the hydrodeoxygenation and decarbonylation reactions need hydrogen to remove the oxygen atom, raising the temperature can help make hydrogen more soluble in the liquid mixture. According to Table, as pressure increased, the yield of the biodiesel fuel range in the liquid product decreased while the yield of the light fraction and the bio-jet range increased. The different routes to form C17 (DCO/DCO_2_) and C18 (HDO) paraffin may be the main reason for the observed opposite effect of pressure on biodiesel yield^45^.

**Table 4:**
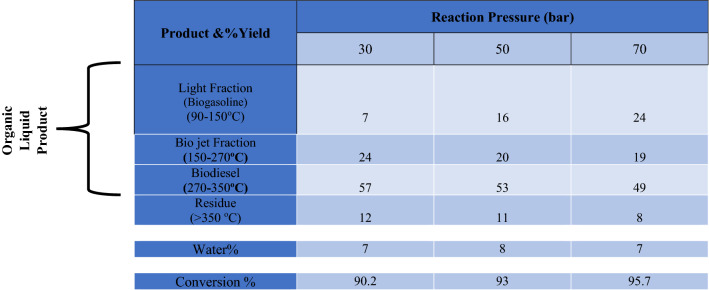
Effect of Operating Pressure on Hydro-conversion of WCO over CoAl_2_O_4_ Nanoparticles. (Temperature 400 °C, LHSV: 1 h^-1^).

Given that hydrogen consumption is proportional to H_2_ pressure, higher H_2_ pressure should increase the amount of adsorbed hydrogen on the surface-active sites, promoting the HDO reaction while inhibiting the DCO/DCO_2_ reaction, suggesting that high pressure is more beneficial to the HDO reaction^45^. By scavenging the deactivating surface species, H_2_ may be necessary to prevent catalyst deactivation^45,46^. In addition, H_2_ is necessary to separate the glycerides from the fatty acids in preparation for subsequent reactions^47–49^.

#### Effect of liquid hourly space velocity

Space velocity was discovered to have an erratic impact on the conversion of WCO for catalytic cracking studies, and since it controls the time of feed into the catalyst, the LHSV is an important operating factor for managing catalyst lifetime and functionality^39,40,50^. 1, 1.5, and 2 h^-1^ were the three different LHSV values that were tested. The distributions of the hydrocracking products of the organic liquid are summarized in Table 5 below. The table shows that the maximum WCO conversion occurred at LHSV of 1 h^-1^ at 400 °C and 50 bar of hydrogen pressure, while the conversion decreased as space velocity increased to 1.5 and 2 h^-1^, which is consistent with some other works^35,51^**.**

**Table 5:**
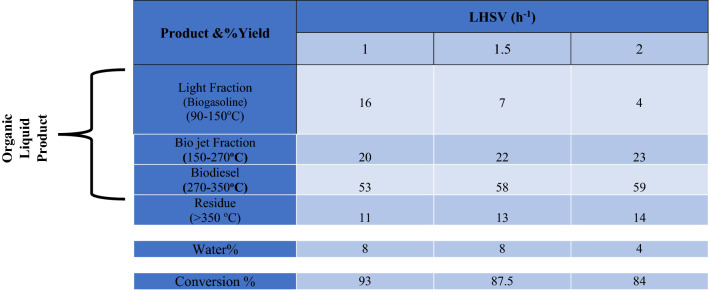
Effect of Liquid Hourly Space Velocity of WCO over CoAl_2_O_4_ Nanoparticles. (Temperature 400 °C, Pressure 50 bar).

The elevated LHSV could prevent cracking reactions. The light fraction hydrocarbon yield decreased as LHSV increased, whereas the fraction yields for bio-jet and biodiesel increased. This could imply that at higher LHSVs, hydrodeoxygenation and subsequent cracking of the n paraffins did not occur due to insufficient residence time. Increasing the LHSV can lead to a relatively higher kerosene/diesel fraction yield because it can suppress cracking Table 5. This fact seems to be in line with other earlier discoveries^52^.

#### Optimum reaction conditions

The best -operating conditions for hydrocracking units must be established due to the dynamic nature of production requirements. A suitable catalyst and the ideal operating conditions necessary for a successful hydrocracking process have been the subject of numerous attempts.

According to the findings of Tables 3, 4, and 5, the best conditions for producing a high yield of light product with a hydrocarbon content of 16 percent (bio-gasoline) and a high percentage yield of 20 percent and 53 percent biodiesel fuel fractions in this study were 400°C, 50 bar, and 1 hour. Since gasoline is a mixture of many atom kinds, its composition is unknown. A rough analysis of the composition is as follows: “15% C4–C8 straight-chain alkanes, 25–40% C4–C10 branched alkanes, 10% cycloalkanes, less than 25% aromatics (benzene less than 1.0%), and 10% straight-chain and cyclic alkenes”^53,54^. Kerosene, on the other hand, has a pretty complicated chemical makeup, consisting of a complex mixture of “paraffin (55.2%), naphthene (40.9%), and aromatic hydrocarbons (3.9%).

**Figure 11:**
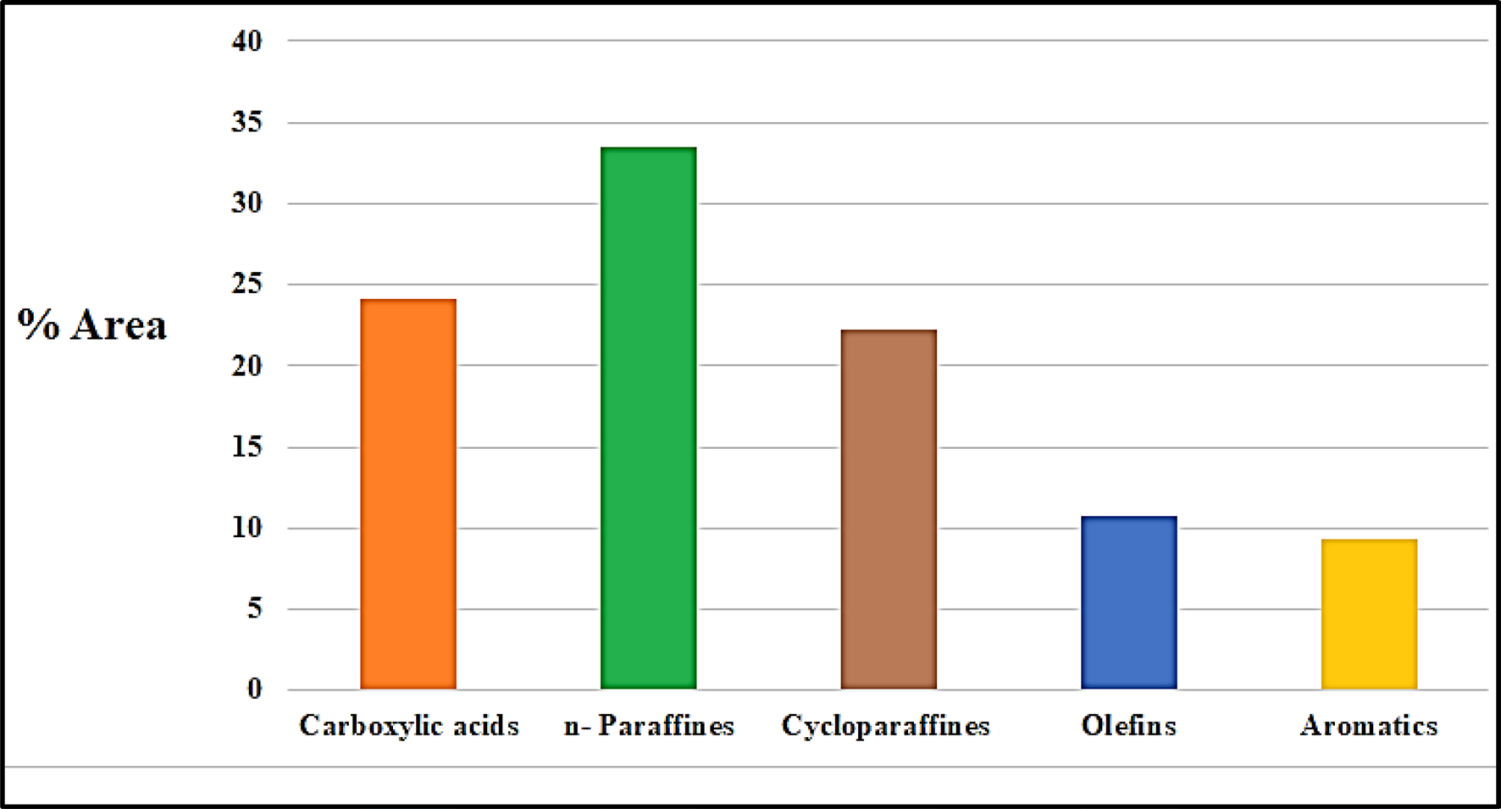
Liquid Product Composition of a Selected Sample. (Temp. 400 °C, 50 bar, LHSV: 1 h^-1^).

Hydrocarbons found in kerosene range in chain length from 11 to 13 carbons”^55^. To determine the composition of a sample obtained under the specified reaction conditions, GC-Ms analysis was used and the results were illustrated in Figure 11.

The abundance of carboxylic acid which makes up 24.17 percent of the total area, indicates that the catalytic hydrocracking process has broken down the triglycerides in raw WCO into saturated fatty acids**.** This demonstrated that the unsaturated fatty acids in the raw material had been broken down into saturated fatty acids. The contribution of cobalt aluminate nanoparticles to the cracking process resulted in the conversion of unsaturated carboxylic acid compounds to saturated carboxylic acid through hydrogenation. The barrier between C=O bonds can be lowered by the presence of (Co)^53,56^.

At a reaction temperature of 400 °C, which was suitable for cracking activity, the decarbonylation reaction took place successfully after the formation of the saturated fatty acid ^57,58^**.** In addition, the formation of hydrocarbons like olefin (10.74 percent by area), n-paraffin (33.53 percent by area), cycloparaffin (22.26 percent by area), and aromatics (9.30 percent by area) shows that double bonds in long chain fatty acids were successfully broken in a safe amount. The dehydrogenation of n-paraffin produced the olefin compound^59,60^.

**Figure 12:**
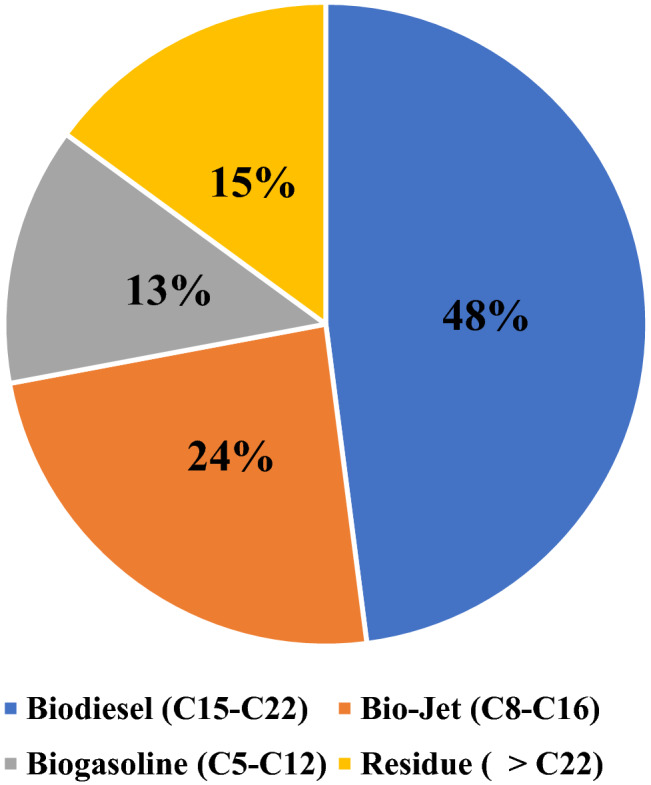
Hydrocarbon Contents of a Selected Sample liquid Product. (Temp. 400 °C, 50 bar, LHSV: 1 h^-1^).

Figure 12 showed that the bio-range hydrocarbons (C15-C22), which make up 47.96% of the total area, are the most prevalent portion of biodiesel. Another fraction was the bio-jet hydrocarbon range (C8-C16), which had a 24.09 percent area, and the bio-gasoline range (C5-C12), which had a 13 percent area. When free fatty acids were cracked into a component of the biofuel at a reaction temperature of 400 °C, 50 bar, and 1 h^-1^ LHSV, the results of the analysis of the GC-MS largely agreed with the end product of the fractional distillation process of the sample.

Compared to previous research findings demonstrated in Table 6, it is noted from the results that the cobalt aluminate Nano catalyst (CoAl_2_O_4_) in the present study had a high potential to help advance the thermo catalytic cracking in a continuous high-pressure reactor, where it led to a high percentage yield of liquid biofuels distributed as a light product (bio-gasoline) of 16% along with high percentage yields of bio-jet and biodiesel fuel fractions of 20% and 53%, respectively. Therefore, it may be said that this catalyst is specifically selective for biodiesel and biojet fuel. As for the % yield of gasoline, it was relatively average, while in another study the percentage of bio-gasoline production exceeded 64% when using ultra-stable Zeolite USY^66^. On the other hand, NiMo/γ-Al_2_O_3_- β-zeolite was more selective to produce diesel range hydrocarbons, reaching 98% by hydrodeoxygenation process at 350 °C and 5 MPa^68^. At the same time, when Zn/HZSM-5 is used as a catalyst for the conversion of palm oil into biofuel by thermo catalytic cracking in micro reactor, it is noted that the yield of bio-gasoline and bio-jet did not exceed 2.54%. and 1.67%, respectively, while the percentage yield of biodiesel range fuel was 23.97%^62,70^.

**Table 6:** Previous Studies Findings in the Catalytic Conversion of Vegetable Oils into Liquid Biofuels.

Catalyst type	Feedstock	Product	% Yield	Conditions	Technique	Reference
CoAl_2_O_4_ nanoparticles	WCO	Biogasoline Range Bio-jet Range Biodiesel Range	16% 20% 53%	400 °C, 50 bar, and 1 h^-1^ (LHSV)	Thermo catalytic cracking in Continuous High-Pressure Reactor	Present work
Cobalt-impregnated carbon catalysts (Co-carbon)	WCO	Liquid Oil Fraction	32,65%	500 °C	Thermo catalytic cracking	Prabasari, I. G., Sarip, R., & Rahmayani, S. (2019). Catalytic cracking of used cooking oil using cobalt-impregnated carbon catalysts. *Makara Journal of Science*, *23*(3), 7^61^
Zn/HZSM-5	Palm Oil	Biogasoline range Bio-jet range Biodiesel range	2.54% 1.67% 23.97%	400 °C	Thermo catalytic cracking in micro reactor	Widayat, W., Saputro, S. A., Ginting, E. M., Annisa, A. N., & Satriadi, H. (2017). Biofuel production by catalytic cracking method using Zn/HZSM-5 catalyst. *ARPN Journal of Engineering and Applied Sciences*, *12*(22), 6347–6351^62^
HZSM-5	Palm oil	Biogasoline range Bio-jet range Biodiesel range	17.11% 14.89% 10.86%	450 °C	Fixed bed microreactor	Roesyadi, A., Hariprajitno, D., Nurjannah, N., & Santi, D. S. (2013). HZSM-5 catalyst for cracking palm oil to gasoline: A comparative study with and without impregnation. *Bulletin of Chemical Reaction Engineering & Catalysis*, *7*(3), 185^63^
Ni/HZSM-5	Palm oil	Biogasoline range Bio-jet range Biodiesel range	17.55% 13.48% 5.84%	450 °C		
Cu/HZSM-5	Palm oil	Gasoline Kerosene Diesel	18.05% 13.30% 5.72%			
Zirconium oxide (ZrO2)	WCO	Organic liquid product (OLP)	83%	475 °C,		Wako, F. M., Reshad, A. S., Bhalerao, M. S., & Goud, V. V. (2018). Catalytic cracking of waste cooking oil for biofuel production using zirconium oxide catalyst. Industrial Crops and Products, 118, 282–289^64^
Nanocrystalline zeolite Y catalyst	Palm WCO	Organic Liquid Product (OLP)	46.5% (33.5 wt% gasoline fraction yield)	458 °C,	Catalytic cracking	Taufiqurrahmi, N., Mohamed, A. R., & Bhatia, S. (2011). Production of biofuel from waste cooking palm oil using nanocrystalline zeolite as catalyst: process optimization studies. Bioresource technology, 102(22), 10,686–10,694^65^
Ultra-Stable Zeolite USY	WCO	Gasoline-based fuels	> 64%	430 °C	Catalytic cracking	Li, L., Ding, Z., Li, K., Xu, J., Liu, F., Liu, S., … & Ge, X. (2016). Liquid hydrocarbon fuels from catalytic cracking of waste cooking oils using ultrastable zeolite USY as catalyst. *Journal of Analytical and Applied Pyrolysis*, *117*, 268–272^66^
Strontium Oxide (SrO) loaded اierarchical Y-zeolite	WCO	Bio-oil	55.3%	550 °C	Pyrolysis	Dada, T. K., Islam, M. A., Kumar, R., Scott, J., & Antunes, E. (2022). Catalytic co-pyrolysis of ironbark and waste cooking oil using strontium oxide-modified Y-zeolite for high-quality bio-oil production. *Chemical Engineering Journal*, *450*, 138,448^67^
NiMo/γ-Al2O3- β-zeolite	Pretreated-WCO	Diesel range hydrocarbons	98% of diesel range hydrocarbons which could be further isomerized to biojet fuel	350 °C at 5 MPa	Hydrodeoxygenation	Li, Z., Huang, Z., Ding, S., Li, F., Wang, Z., Lin, H., & Chen, C. (2018). Catalytic conversion of waste cooking oil to fuel oil: Catalyst design and effect of solvent. *Energy*, *157*, 270–277^68^
Zeolite Meso-Y	WCO	Biojet fuel	52%	400 _C	Hydro catalytic cracking	Li, T., Cheng, J., Huang, R., Zhou, J., & Cen, K. (2015). Conversion of waste cooking oil to jet biofuel with nickel-based mesoporous zeolite Y catalyst. *Bioresource technology*, *197*, 289–294^69^
KOH catalyst based on calcined cow bone	WCO	Biodiesel	99.56%	63.53 °C	Transesterification in a T-shaped microreactor	Aghel, B., Mohadesi, M., Razmehgir, M. H., & Gouran, A. (2022). Biodiesel production from waste cooking oil in a micro-sized reactor in the presence of cow bone-based KOH catalyst. *Biomass Conversion and Biorefinery*, 1–15^70^

#### Physical and chemical properties of liquid product biofuels

Some of the properties of the obtained biofuel cuts' fractions were displayed in Tables 7 and 8. The produced fraction biodiesel sample underwent a qualitative test for properties including density, sulfur content, flash point, kinematic viscosity, water and sediment content, pour point, and copper corrosion strip. The results are shown in Table 7. The ASTM standards were found to be met or exceeded by the biodiesel fuel's properties.

**Table 7:** Physical and Chemical Properties of Biofuels (Biodiesel) in Diesel Fraction Range (270–370 °C).

Properties	Standard test methods^71–73^	Results
Density @ 15 °C; (g/cm^3^)	ASTM D-4052 (0.8–0.9)	0.8481
Sulfur Content; (%wt.)	ASTM D-5453 (0.02% max.)	0.0015
Flash Point (P.M.C.C); (°C)	ASTM D-93 (> 130 min.)	61
Pour Point; (°C)	ASTM D-97	0
Kinematic Viscosity @ 40 °C ; (cSt)	ASTM D-445 (1.9–6)	5.113
Water and Sediment Content; (%vol)	ASTM D-2709 (0.05 max.)	Nil
Copper Corrosion Strip @ 50 °C/3 h	ASTM D-130 (No.3 max.)	1A

**Table 8:** Physical and Chemical Properties of Biofuels in Jet Fraction (150–270 °C).

Test	Standard Test Method^71,72,74^	Results
Density @ 15 °C ; (g/cm^3^)	ASTM D-4052 (0.775–0.840)	0.8322
Flash point °C	IP 170 (38 min.)	44
Gum content mg/100 ml	ASTM D381 (7 max.)	4.2
Freezing point °C	ASTM D 7153 (− 47 max.)	− 80

A specific temperature range (150–270 °C) was used to analyze the properties of the bio-jet fraction Table 8. In this region of the distillation range, the investigated values for the flash point (44 °C), gum content (4.2 mg/100 ml), and freezing point (− 80 °C) all met the ASTM-D7566 international specifications for bio-jet fuel.

## Conclusions

Biofuels made from WCO are renewable and a great alternative to fuels made from petroleum. Among the various production techniques, HDO is a desirable option. In this study, CoAl_2_O_4_ nanoparticles were prepared and characterized then used as a catalyst in catalytic cracking processes to turn WCO into biofuel. Process performance is influenced by some factors, which alter product yield and specifications. The yield and product quality were significantly impacted by the variation in cracking temperature. The reaction temperature, pressure, and LHSV were three key hydrocracking operating parameters that were examined. According to the findings, WCO catalytic hydrocracking creates fuel with a chemical makeup resembling petroleum-based fuel. As temperature and pressure rose, the proportion of bio-jet/biodiesel fell while that of bio-gasoline rose.

According to the results, 400 °C, 50 bar, and 1 h^−1^ are the ideal conditions for producing a high yield of light product (bio-gasoline) of 16% along with high percentage yields of bio-jet and biodiesel fuel fractions of 20% and 53%, respectively. Thus, it is possible to use the prepared nanocatalyst in the production of biofuel from raw materials locally, which may reduce the import cost. It is planned to conduct a subsequent study on reusing the catalyst several times.

## Data Availability

The data sets generated and analyzed during the current study are not publicly available because they belong to a not yet awarded thesis but are available from the corresponding author on reasonable request.
